# Poly(Lactic Acid) Composites with Lignin and Nanolignin Synthesized by In Situ Reactive Processing

**DOI:** 10.3390/polym15102386

**Published:** 2023-05-19

**Authors:** Sofia P. Makri, Eleftheria Xanthopoulou, Miguel Angel Valera, Ana Mangas, Giacomo Marra, Víctor Ruiz, Savvas Koltsakidis, Dimitrios Tzetzis, Alexandros Zoikis Karathanasis, Ioanna Deligkiozi, Nikolaos Nikolaidis, Dimitrios Bikiaris, Zoi Terzopoulou

**Affiliations:** 1 Creative Nano PC, 43 Tatoiou, Metamorfosi, 14451 Athens, Greece; s.makri@creativenano.gr (S.P.M.); a.karathanasis@creativenano.gr (A.Z.K.); i.deligkiozi@creativenano.gr (I.D.); 2 Laboratory of Polymer and Colors Chemistry and Technology, Department of Chemistry, Aristotle University of Thessaloniki, 54124 Thessaloniki, Greece; elefthxanthopoulou@gmail.com (E.X.); nfnikola@chem.auth.gr (N.N.); 3 AIMPLAS, Asociación de Investigación de Materiales Plásticos Y Conexas, Carrer de Gustave Eiffel, 4, 46980 Valencia, Spain; mavalera@aimplas.es (M.A.V.); amangas@aimplas.es (A.M.); gmarra@aimplas.es (G.M.); vruiz@aimplas.es (V.R.); 4 Digital Manufacturing and Materials Characterization Laboratory, School of Science and Technology, International Hellenic University, 14 km Thessaloniki, 57001 N. Moudania, Greece; skoltsakidis@ihu.edu.gr (S.K.); d.tzetzis@ihu.edu.gr (D.T.)

**Keywords:** biobased polymers, poly(lactic acid), lignin, nanolignin, reactive extrusion, reactive processing, in situ polymerization

## Abstract

Poly(lactic acid) (PLA) composites with 0.5 wt% lignin or nanolignin were prepared with two different techniques: (a) conventional melt-mixing and (b) in situ Ring Opening Polymerization (ROP) by reactive processing. The ROP process was monitored by measuring the torque. The composites were synthesized rapidly using reactive processing that took under 20 min. When the catalyst amount was doubled, the reaction time was reduced to under 15 min. The dispersion, thermal transitions, mechanical properties, antioxidant activity, and optical properties of the resulting PLA-based composites were evaluated with SEM, DSC, nanoindentation, DPPH assay, and DRS spectroscopy. All reactive processing-prepared composites were characterized by means of SEM, GPC, and NMR to assess their morphology, molecular weight, and free lactide content. The benefits of the size reduction of lignin and the use of in situ ROP by reactive processing were demonstrated, as the reactive processing-produced nanolignin-containing composites had superior crystallization, mechanical, and antioxidant properties. These improvements were attributed to the participation of nanolignin in the ROP of lactide as a macroinitiator, resulting in PLA-grafted nanolignin particles that improved its dispersion.

## 1. Introduction

The low cost and high versatility of plastic products have made them ubiquitous in everyday life. However, their widespread use has resulted in the generation of large quantities of waste and raised concerns about their environmental, health, and economic impacts. To address this issue, the Global Bioeconomy has suggested the development of bioplastics, which can support a more sustainable plastic life cycle within a circular economy [1]. Poly(lactic acid) (PLA) is one of the most promising biobased polymers to replace conventional oil-based plastics as it is derived from renewable sources such as corn starch, sugarcane, or potato starch. PLA is produced through the fermentation of sugars obtained from these sources, which are then converted to lactic acid. Lactic acid is then either directly polymerized to PLA or transformed to lactide first [2]. High availability and compostability are some of the advantages of PLA [3]. However, PLA suffers from limited mechanical properties, moisture sensitivity, poor antioxidant activity, and low thermal resistance [2].

Lignin is a biopolymer that is isolated from lignocellulosic biomass and is the second most abundant natural polymer after cellulose. When added to polymers, it imparts them antioxidant and antibacterial properties [4,5,6], which makes it a very attractive additive for food packaging, agriculture, and biomedical applications. Lignin has a complex structure and varies in composition and properties depending on its origin and the method used to isolate it [7,8]. Lignin has a highly branched structure that contains functional groups such as hydroxyls, which can form hydrogen bonds with the carboxylic groups of PLA. Furthermore, the aromatic rings present in lignin can form π-π interactions with the carbonyl groups of PLA, resulting in increased interfacial adhesion between the two materials [5]. However, lignin tends to aggregate during melt mixing because of intermolecular interactions, which limits its potential applications. Additionally, the poor interfacial bonding between the lignin particles and the polymer matrix tends to favor particle aggregation and phase separation in the resulting blends [8]. As a result, tensile strength, elongation at break, and impact strength can deteriorate depending on the filler loading [9,10,11,12]. Modification of lignin’s surface functionalities such as esterification, methylation, oxidation, amination, and polymer grafting with ring opening polymerization [13,14,15,16,17,18] can make lignin more compatible with a wide range of polymers, thus enhancing its properties and expanding its potential applications in the field of composite materials [19].

Even though many publications on PLA-lignin composites are available, most of them do not mention the particle size of lignin and disregard its effect on the obtained results. A few studies have demonstrated that size reduction of lignin from the microscale to sub-micron particles helps achieve better reactivity and compatibility with PLA due to the high specific surface area and better dispersion ability, which allows it to bond more effectively with the PLA matrix [5,20,21,22,23,24,25,26,27]. In all cases, the composites were prepared by either melt mixing or solution casting. In our previous study, Makri et al. [28] showed that a uniform distribution of nanolignin in PLA matrix can result in improved mechanical properties of the nanocomposites that were prepared by melt mixing. PLA-nanolignin composite films consistently performed better than their PLA-lignin counterparts due to the finer distribution of nanolignin in the PLA matrix. Boarino et al. [21] grafted PLA on nanolignin before introducing it into PLA with solution casting, which improved their dispersion, UV blocking, and antioxidant properties. However, the fact that a two-step process was necessary and the use of harmful solvents make this approach time-consuming, not cost-effective and not environmentally friendly to scale up.

When compared to melt mixing, the in situ polymerization method to prepare nanocomposites allows better dispersion of the additives by forming covalent bonds between lignin and PLA [29]. Melt mixing, on the other hand, allows for the fast and large-scale production of nanocomposites since this method is predominantly used in the industry. To take advantage of the features of both these methods, we combined in situ ring opening polymerization (ROP) with reactive processing (to simulate reactive extrusion (REX) on a smaller scale) to prepare PLA composites with lignin and nanolignin. To do so, both the monomer (L-lactide) and the additives (lignin or nanolignin) were fed into a torque rheometer, which simulates melt extrusion at bench scale, where in situ polymerization took place. This approach allows the fast preparation of the composites in one step without the use of solvents.

In summary, we investigated the preparation of PLA composites with 0.5 wt% kraft lignin at the micro- and nanoscales using two different methods: (i) melt compounding and (ii) one-pot in situ polymerization with reactive processing. Melt compounding was performed for comparison since it is the conventional approach. To explore the effect of lignin particle size, commercially available kraft lignin with a diameter of 2.7 μm and nanolignin with a diameter of 650 nm were utilized. Subsequently, the prepared composites were pressed into films to evaluate their properties. GPC and NMR techniques were employed to analyze the resulting composites, and their thermal properties and crystallization were investigated using differential scanning calorimetry (DSC). The dispersion of lignin and nanolignin in the PLA composites was assessed using SEM and optical microscopy. Finally, the mechanical, antioxidant, optical, and water contact angles of the composites were evaluated.

## 2. Materials and Methods

### 2.1. Materials

Polylactic acid (PLA) Ingeo^TM^ Biopolymer 2003D (D-isomer 4%, M_n_ = 114,300 g/mol and M_w_ = 181,700 g/mol, specific gravity 1.24, MFR 6 g/10 min at 210 °C) from Natureworks (Minneapolis, MN, USA) was kindly donated by Plastika Kritis S.A., Irakleio, Greece. L-Lactide (LA) Purlact^®^ B3 (purity 99% *w*/*w*, stereochemical purity in L-isomer 95% (*w*/*w*)) was purchased from Corbion N.V. (Gorinchem, The Netherlands). Tin(II) 2-ethylhexanoate (Sn(Oct)_2_) (purity > 92.5%) and triphenyl phosphine (TPP) (purity > 98.5%) were purchased from Merck KGaA (Darmstadt, Germany). Raw kraft lignin (BioPiva300) was purchased from UPM (Helsinki, Finland). Kraft nanolignin was kindly provided by Creative Nano PC (Athens, Greece). Before use, the fillers lignin (L) and nanolignin (NL) were first dried overnight at 110 °C under vacuum. 2,2-diphenyl-1-picrylhydrazyl (DPPH, 95%) and all other chemicals used were purchased from Sigma-Aldrich (St. Louis, MO, USA).

### 2.2. Preparation of Composites with Melt Compounding

PLA 2003D (extrusion grade) and its composites with lignin and nanolignin were prepared by melt mixing in a Brabender^®^ Plasti-Corder^®^ (Duisburg, Germany) Lab-Station torque rheometer. Both PLA and the appropriate amount of filler for a final content of 0.5 wt% were fed into the rheometer in the form of a pre-mix with the help of a pneumatic ram and mixed at 180 °C, screw speed 50 rpm, for 5 min. Films were prepared by compression molding using an Otto Weber Type PW 30 hydraulic press connected with an Omron E5AX Temperature Controller (Kyoto, Japan) at a temperature of 180 ± 5 °C and a pressure of 100 mbar. After melt pressing, the films were cooled rapidly at room temperature. 

### 2.3. Preparation of Composites with In Situ Reactive Processing

PLA and its composites with lignin and nanolignin were prepared by reactive processing in a Brabender^®^ Plasti-Corder^®^ Lab-Station torque rheometer. The ROP of lactide was catalyzed by Sn(Oct)_2_ in a lactide/Sn(Oct)_2_ molar ratio of 1000/1. Triphenyl phosphine (TPP) was used as a co-catalyst in an equimolar amount with Sn(Oct)_2_ [30]. For the preparation of 0.5 wt% PLA/L and PLA/NL composites, the appropriate amount of filler was premixed with L-lactide and the catalyst system and fed to the rheometer. The temperature was set at 180 °C, the screw speed was 50 rpm, and N_2_ gas was circulated in the mixer. The reaction times were 8.5 min for neat PLA, 19.5 min for PLA ROP L, 13 min for PLA ROP NL C, 18 min for PLA ROP L, and 11 min for PLA ROP L C. The abbreviations are explained in Table 1.

### 2.4. Characterization Methods

#### 2.4.1. Characterization of Lignin and Nanolignin

The average particle size of raw and nano-scale kraft lignin was measured via dynamic light scattering (DLS) on a Litesizer 500 instrument (Anton Paar, Graz, Austria). The powders were dispersed and ultrasonicated in deionized (DI) water at a concentration of 100 ppm for 5 min prior to measurement. The hydrodynamic diameter was around 2.7 μm (polydispersity index, PDI = 28%) for raw lignin and 651 nm (PDI = 23%) for nanolignin. Particle size distribution curves for both samples are presented in Figure S1a. The thermal stability was evaluated with TGA (Figure S1b), where it was confirmed that both L and NL are thermally stable upon heating in air up to 180 °C.

#### 2.4.2. Characterization of PLA Composites with Lignin and Nanolignin

Gel permeation chromatography (GPC) was carried out using a high-performance liquid chromatograph (HPLC—Waters 1515) with a photodiode array detector (Waters 2998 PDA) and a gel permeation column (GPC—Tosoh Bioscience, TSKgel GMHHR-H, 5 µm, 7.8 mm × 300 mm). Ten polystyrene (PS) standards with molecular weights in the range of 418 to 2,100,000 g/mol were used for calibration. The samples were first dissolved in CHCl_3_ at a 4 mg/mL concentration and passed through a 0.22 µm PTFE microfilter to remove any solid residue. A volume of 30 µL of filtered polymer was injected at 1 mL/min at room temperature. The calculation of the molecular weight distribution of the samples was carried out using Empower 4 software from Waters.

Proton nuclear magnetic resonance (^1^H-NMR): The ^1^H was recorded at 303 K on a Bruker AV300 spectrometer after dissolving 10 mg of polymer in 600 µL of deuterated chloroform. The NMR spectra were calibrated to the residual solvent signal of CDCl_3_ δ (^1^H) = 7.26 ppm.

The intrinsic viscosity of the produced polyesters was measured with an Ubbelohde viscometer (Schott Gerate GMBH, Hofheim, Germany) at 25 °C using chloroform as solvent. The sample was heated in the solvent mixture at 50 °C for 20 min until complete dissolution. After cooling, the solution was filtered through a disposable Teflon filter to remove possible solid residues. The calculation of the intrinsic viscosity value of the polymer was performed by applying the Solomon–Ciuta Equation (1) to a single point measurement:

(1) $$\left[\eta \right]=\frac{{\left[2\left\{\frac{t}{t_{0}}-ln\left(\frac{t}{t_{0}}\right)-1\right\}\right]}^{\frac{1}{2}}}{c}$$

where *c* is the solution concentration, *t* is the flow time of the solution, and *t*_0_ is the flow time of the solvent. The experiment was performed three times, and the average value was estimated.

The morphology of cryofractured cross sections of the samples was studied with a JEOL (Tokyo, Japan) JSM 7610F field emission scanning electron microscope (SEM) operating at 5 kV. Photographs of thin films were also captured using a Jenoptik (Jena, Germany) ProgRes GRYPHAX Altair camera attached to a ZEISS (Oberkochen, Germany) SteREO Discovery V20 microscope and the Gryphax image capturing software.

Differential scanning calorimetry (DSC) analysis was performed using a PerkinElmer Pyris Diamond DSC differential scanning calorimeter (Solingen, Germany) calibrated with pure indium and zinc standards. The system included a PerkinElmer Intracooler 2 (Solingen, Germany) cooling accessory. Samples of 5 ± 0.1 mg sealed in aluminum pans were used to test the thermal behavior of the polymers. The crystallinity degree (X_c_) was calculated with Equation (2):

(2) $$\;Xc_{\;}(\%)=\left(\frac{\Delta H_{m}-\Delta H_{cc}}{\Delta H_{f}^{0}-\frac{1-wt\%\;additive}{100}}\right)\times 100$$

where *ΔH_m_*, $\Delta H_{cc}$, $\Delta H_{f}^{0}$ are the experimental melting enthalpy, the cold-crystallization enthalpy, and the theoretical heat of fusion of 100% crystalline PLA ($\Delta H_{f}^{0}=$93 J/g), respectively.

A polarizing light microscope (Nikon Optiphot-2, Tokyo, Japan) equipped with a Linkam THMS 600 heating stage, a Linkam TP 91 control unit, and a Jenoptic ProgRes C10Plus camera with Jenoptik Gryphax® V2.2 CapturePro software was utilized for PLM observations.

Tensile tests were performed using an Instron 3344 dynamometer (Norwood, MA, USA), according to ASTM D882, using a crosshead speed of 5 mm/min. Dumb-bell-shaped tensile test specimens (central portions 5 mm × 0.5 mm thick, 22 mm gauge length) were prepared by compression molding in a thermopress at 180 °C, cooled rapidly, and cut in a Wallace cutting press. At least five measurements were conducted for each sample, and the results were averaged to obtain the mean values of elastic modulus, tensile strength at yield and breakpoint, and elongation at break.

The nanoindentation tests were performed using a DUH-211S Shimadzu device (Kyoto, Japan) with a force resolution of 0.196 μN. The tests utilized a diamond triangular-tip Berkovich indenter with an angle of 65° and a tip radius of 100 nm. The hardness values were calculated based on the indentation depth and the predetermined applied force. The calculation of the elastic modulus and hardness was based on the Oliver and Pharr method [31] and previous work [32,33,34,35,36]. The maximum applied force was 20 mN and was achieved at a rate of 1.46 mN/s. In order to calculate the nanomechanical properties, five measurements were carried out at different locations for each experiment, and the average values were reported. Due to the material’s viscoelastic nature, a dwell time of 3 s was implemented to allow sufficient time at peak load for the creep effects to saturate. The additional depth induced during the dwell time at constant load was recorded to provide insight into the creep response of the material. A finite element analysis (FEA) process has been developed to fit the nanoindentation test curves and extract the stress–strain behavior of the specimens. The interface between the indenter and the surface of the sample was simulated with contact elements and assumed to be frictionless. The nanoindentation experiments have been computationally generated, considering the simulation of the loading stage of the indenter penetrating the surface. Other works [32,33,34,35,36,37,38,39,40,41,42] have shown that kinematic hardening leads to rapid convergence in the corresponding FEA calculations, so this method was utilized in the developed curve-fitting procedure.

The antioxidant activity of two PLAs and PLA-L/NL composites was evaluated by monitoring the reduction rate of the DPPH radical in the antioxidant’s (L/NL) presence via UV-Vis spectroscopy. This technique measures the ability of a substance to scavenge free radicals by observing the decrease in absorbance of a DPPH solution after incubation with the test sample. A 0.079 mM DPPH solution in EtOH was prepared and stored in the dark for 16 h at room temperature. The prepared films with the same dimensions (1 cm × 1 cm) were immersed in 3 mL of the DPPH/EtOH solution at room temperature and kept in the dark. The composites’ antioxidant capacity was determined by measuring the absorption decay at 517 nm at regular time intervals via UV-Vis. The residual DPPH content in the solution was calculated using Equation (3):

Residual DPPH content (%) = 100 − 100 (A_0_ − A_1_/A_0_) (3)

where A_0_ is the absorbance of the control sample and A_1_ is the absorbance in the presence of the films.

Diffuse Reflectance Spectroscopy (DRS) was used to measure the PLA-based composites using an Agilent Carry 60 spectrophotometer (Agilent Technologies, Santa Clara, CA, USA) equipped with a Harrick VideoBarrelino DRA fiber optic coupler (Pleasantville, NY, USA) between 200 and 800 nm. For each composite film, 100% transmittance was normalized at 800 nm. The baseline correction was carried out in BaSO_4_ standard.

Water contact angle measurements were performed using an optical tensiometer, One Attention (Biolin Scientific, Espoo, Finland). The sessile water droplet method was used to investigate the hydrophilicity of the PLA-based films as a result of the addition of L and NL. Measurements were performed in triplicate.

### 2.5. Statistical Analysis

Where applicable, statistical analysis was performed with a one-way ANOVA with a post hoc Tukey test. The software used was GraphPad Prism 6. A *p*-value of < 0.05 was considered statistically significant.

## 3. Results

### 3.1. Synthesis and Chemical Structure of PLA Composites with Lignin and Nanolignin

When the ROP of lactide is performed in the presence of L with either metal alkoxide or organocatalysts, the PLA chains are grafted onto L [14,15,17,18,20,21]. The grafted PLA chain length is controlled by both the amount of L and the M_n_ reducing as the amount of L increases [13].

PLA/L and PLA/NL composites with 0.5 wt% filler were prepared through two different methods: melt compounding using commercial PLA and in situ ROP by the reactive processing of L-lactide. The samples prepared and their abbreviations are shown in Table 1. The temperature of 180 °C was chosen based on previous work, as it resulted in polymers with a higher molecular weight than the ones obtained through ROP at 190 °C [37], and the lignin content of 0.5 wt% was chosen after preliminary tests showed that when adding ≥1 wt% of either lignin or nanolignin in the reactive processing of L-lactide, the molecular weight decreased drastically.

**Figure 1:**
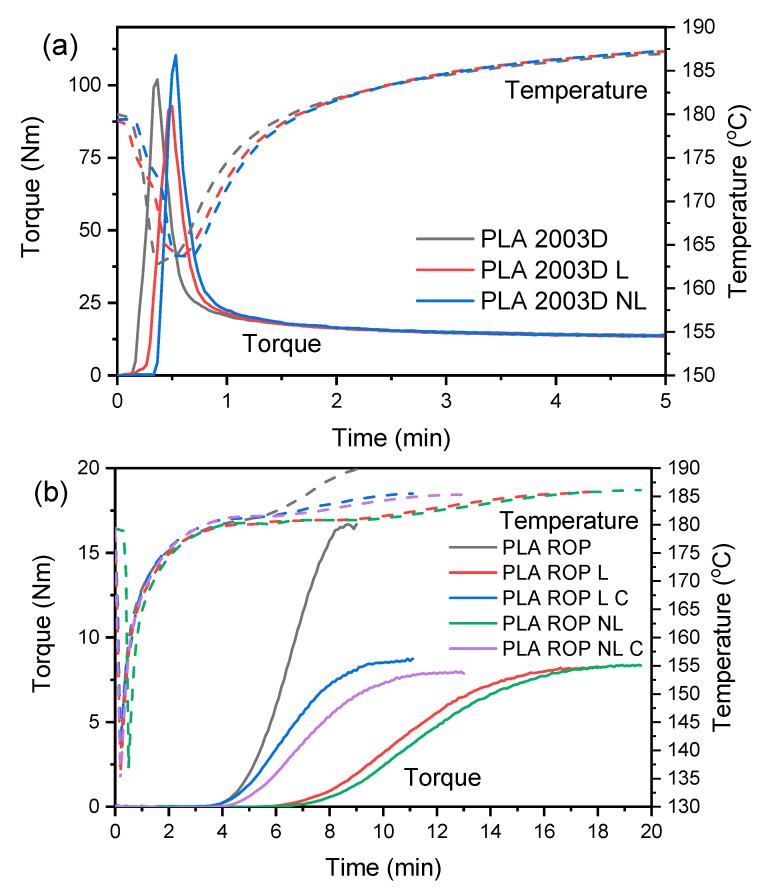
Torque curves of (**a**) the melt compounding of PLA 2003D and (**b**) the ROP of PLA in the presence of lignin and nanolignin.

Torque monitoring during melt compounding allowed the evaluation of the processing stability of PLA 2003D containing L and NL (Figure 1a). A sharp increase in torque occurred because of the loading of the polymer pellets in the rheometer, accompanied by a decrease in temperature. After the loading peak, the polymer melted and the torque decreased due to the effects of shear forces and temperature. Finally, the torque stabilized after 1.5 min, and the temperature reached the set value of 180 °C. The stabilized torque values are the same between PLA 2003D and its composites with L or NL, indicating that no degradation occurred during processing. When melt-processing PLA at 170 °C or 190 °C for longer than 5 min, degradation was observed via a reduction in the torque, which was more pronounced for PLA in the presence of 10 or 25 wt% lignin [38]. In this work, degradation was avoided by using a small amount of lignin and by drying both PLA and lignin to remove moisture, which can have a detrimental effect on polymer degradation.

Torque was also monitored during the reactive processing of PLA (Figure 1b). In all polymers, there is an induction time before torque starts increasing (i.e., before polymerization begins), which is about 4 min. Neat PLA reached the maximum torque value after 8.5 min, while all composites needed more time to polymerize. The addition of NL decelerated the polymerization of PLA more than L, needing 19.5 min to reach maximum torque. By doubling the catalyst level (PLA ROP L C and PLA ROP NL C), the polymerization time was notably reduced (from 19.5 min to 13 min for NL and from 18 min to 11 min for L) without considerably affecting the final torque values.

The molecular weight of the prepared PLA composites was estimated with GPC, and the results are shown in Figure S2 and Table 2. To corroborate the M_n_ values calculated by GPC, intrinsic viscosity [η] was also measured. [η] values decreased in the presence of both L and NL, which was expected because the hydroxyl end groups of lignin act as initiation sites, deactivating the catalyst [37]. When the catalyst amount was doubled (samples PLA ROP L C and PLA ROP NL C), the [η] had a decreasing trend, but the difference was too small to consider it significant. M_n_, on the other hand, increased in all composites in comparison with PLA, a discrepancy with both torque and [η] values, which are values affected by all chain lengths simultaneously, while GPC can detect the different chain lengths and express them as PDI. More specifically, PLA ROP had a M_n_ = 64,000 g/mol and a large PDI of 2.73. The M_n_ of PLA ROP L and PLA ROP L C slightly increased, while their PDI was considerably larger. With the addition of NL, peculiarities were noticed in the GPC curves. The molecular weight distribution was bimodal (Figure S2), with one peak corresponding to very high M_n_ and low PDI and the other with low M_n_ and much higher PDI. The first could arise from PLA grafted to NL, with NL acting as an initiation site, and the second could arise from PLA initiated by traces of moisture. The large M_n_ peak of PLA grafted to NL is likely a result of the large hydrodynamic volume of the PLA chains on each NL particle’s surface. Such a phenomenon was observed also for other PLA nanocomposites with fillers containing many hydroxyl groups [39], as well as lignin, where a star-like structure with a lignin core and polymer particles around it was reported [40]. This phenomenon was not observed in the PLA–ROP–L composites, where M_n_ decreased as predicted, but only in the NL composites. This can be attributed to the smaller particle size, which increased the specific surface and thus the number of hydroxyl groups available to initiate polymerization. The smaller peaks in larger retention times can be associated with free monomers and impurities, and the last peak is attributed to the solvent.

The amount of unreacted lactide was quantified from the relative peak areas of the monomer and polymer methine quartet at 5.03 ppm and 5.16 ppm (A_CH, L_, and A_CH, P_) from the ^1^H-NMR spectra of the polymers (Figure S3) [41]. The calculated lactide content is reported in Table 2. All composites contain less free lactide than neat PLA, either because the number of hydroxyls available to initiate the polymerization is larger or because the longer polymerization time helped to achieve higher lactide conversion as well as partially remove unreacted lactide through the venting port of the mixing chamber.

### 3.2. Morphology and Dispersion

**Figure 2:**
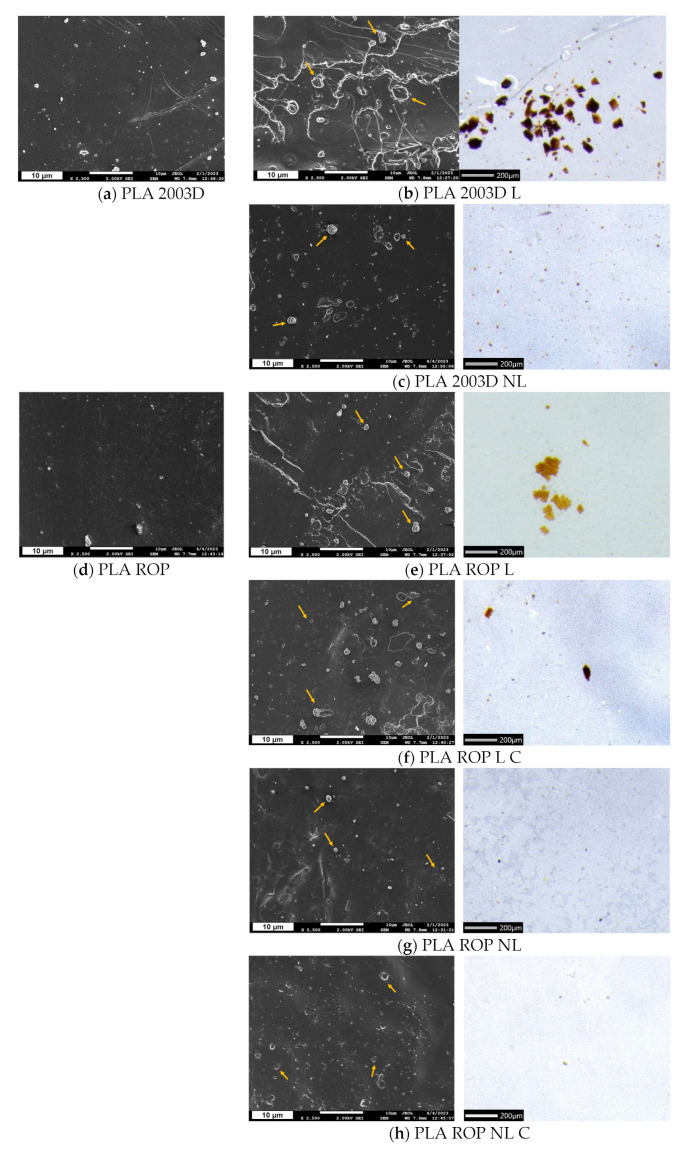
SEM images of cryo-fractured cross sections and stereoscope images of thin films of (**a**) PLA 2003D, (**b**) PLA 2003D L, (**c**) PLA 2003D NL, (**d**) PLA ROP, (**e**) PLA ROP L, (**f**) PLA ROP L C, (**g**) PLA ROP NL, and (**h**) PLA ROP NL C.

The dispersion of L and NL in the different PLA composites was examined with both SEM (×2500) and optical microscopy (×50) (Figure 2). The size difference between L and NL was obvious in the optical microscopy images. When directly added to PLA 2003D with melt compounding, big particles of lignin were detected (Figure 2b), with sizes ranging from 10 μm to 110 μm. When comparing the optical microscope images of the samples PLA 2003D L and PLA ROP L/PLA ROP L C, the latter had fewer and smaller aggregates, a first indication that in situ polymerization with ROP already improves dispersion and prevents aggregation of lignin to a degree. PLA 2003D NL had visible lignin particles with sizes up to ~14 μm and a lot of smaller particles that could not be measured. NL particles were even smaller and had fewer aggregates in the samples prepared by ROP, namely PLA ROP NL and PLA ROP NL C. In the SEM images of samples with L (PLA 2003D L, PLA ROP L, and PLA ROP L C), some particles with various sizes, but always at the microscale, that could be lignin were observed (indicated with arrows). In Figure 2c,g,h, the surfaces of the samples with NL are presented, and there the particles were more difficult to identify because of their smaller size. It was thus confirmed that the NL particles retained their smaller size in the composites. Only a few aggregates with sizes up to ~1.5 μm were detected on the surface of PLA ROP NL and PL ROP NL C (Figure 2g,h).

### 3.3. Thermal Properties and Crystallization

**Figure 3:**
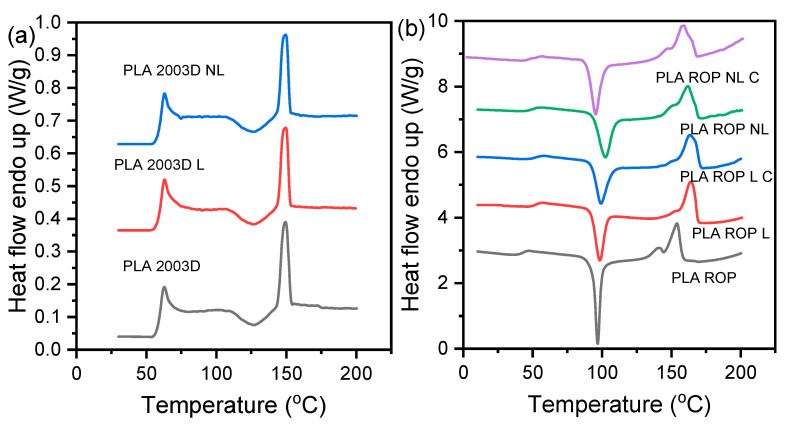
DSC graphs recorded during heating of quenched PLA lignin and nanolignin composites prepared by (**a**) melt compounding and (**b**) ROP.

Lignin is a rigid molecule that can affect the thermal properties of polymers. The thermal characteristics of the prepared composites, exported from the DSC graphs of Figure 3, are shown in Table 3. When added by melt compounding (Figure 3a), neither L nor NL affected the T_g_, T_cc_, or T_m_ of PLA 2003D, which remained about 60 °C, 126 °C, and 150 °C, respectively.

On the other hand, when added via in situ polymerization using reactive processing (Figure 3b), 0.5 wt% of L or NL was enough to affect the thermal transitions of PLA. More specifically, the T_g_ increased from 43 °C to 52 °C with L and to 50 °C with NL. The T_g_ increase was less pronounced in the case of NL, possibly because of the smaller particle size that has a lesser effect on the reduction of free volume and/or the lower M_n_ values (Table 2) of the PLA ROP NL composites in comparison with the PLA ROP L composites. The T_cc_ also increased, with the exception of PLA ROP NL C, indicating the difficulty of the macromolecular chains to reorganize into crystals because of reduced molecular mobility. This was expected because of the covalent bonding of the PLA chain ends on L particles during synthesis. Among the ROP-prepared composites, the sample PLA ROP NL had the highest T_cc_ (102.5 °C), and the sample PLA ROP NL C had the lowest T_cc_ (95.4 °C). It is likely that the larger amount of catalyst might have acted as a heterogenous nucleating agent along with NL [16], causing the decrease in T_cc_ and increase in ΔH_cc_. Finally, the T_m_ of the ROP-prepared composites is higher than that of neat PLA, which is consistent with the increase in T_g_. When adding larger amounts of L or NL, T_g_ was reported to decrease because lignins can act as internal plasticizers [23,24,37].

Cooling scans (Figure S4) revealed limited melt crystallization, with the ΔH_c_ never exceeding 10 W/g. The crystallization peaks were more pronounced in the composites than in neat PLA, which is generally a slow-crystallizing polyester, which could arise from the lower molecular weight of the composites as well as the presence of the particles that help with nucleation. The T_c_ ranges from ~88 to 96 °C, which is within the known range of melt crystallization of PLA. PLA 2003D and its composites had no detectable melt crystallization peaks due to their very high molecular weight.

### 3.4. Mechanical Properties

**Figure 4:**
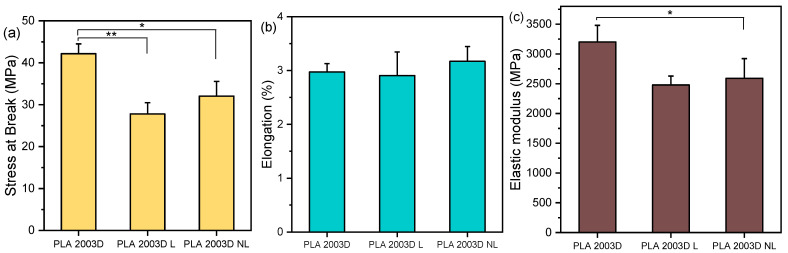
Tensile properties of PLA 2003D with kraft lignin and nanolignin. (**a**) Stress at break, σ_b_, (**b**) Elongation at break, ε_b_, and (**c**) Elastic modulus, E. One-way ANOVA, post hoc Tukey HSD test * *p* = 0.01–0.05, ** *p* = 0.001–0.01.

The mechanical properties of the composites were evaluated by tensile and nanoindentation testing. While it was possible to prepare films with compression molding for the PLA 2003D samples, the ROP samples did not give macroscopically homogenous films suitable for tensile testing. Nanoindentation requires a much smaller area for testing, so the PLA ROP materials were examined with that technique. The stress at break, σ_b_, elongation at break, ε_b_, and elastic modulus, E, of PLA 2003D and its composites are presented in Figure 4. PLA 2003 has σ_b_ = 42 ± 2 MPa, ε_b_ = 3 ± 0.2%, and E = 3200 ± 280 MPa. Both σ_b_ and E decreased after the addition of 0.5 wt% of either L or NL, and ε_b_ remained unaffected, with no statistically significant differences (*p* > 0.05) between PLA 2003D L and PLA 2003D NL. Yang et al. found that the addition of 1 wt% lignin nanoparticles (~50 nm) in PLA with melt mixing slightly improved σ_b_ and E, while 3 wt% reduced them while improving ε_b_, but since there were no comparisons made with micron-sized lignin, one cannot estimate the effect of particle size [25,26]. An amount of 1 and 3 wt% modified NL (200–400 nm) melt mixed in PLA did not affect its tensile properties, while unmodified NL decreased them, in line with our study [42]. In other papers, larger quantities (5–15 wt%) of either micron-sized or submicron lignin had the same effect on the σ_b_, even when it was esterified [43], but also decreased the ε_b_ of PLA composites prepared by melt mixing [24,44]. This deterioration of the tensile properties of melt-mixed PLA lignin composites is attributed to aggregation, the formation of crazes, and a lack of interfacial interactions.

**Figure 5:**
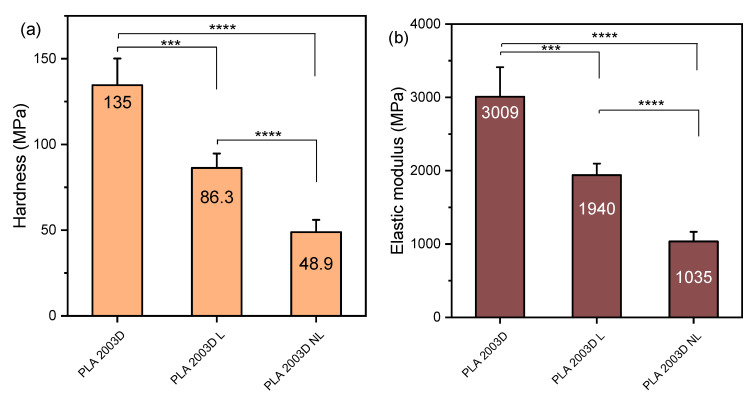

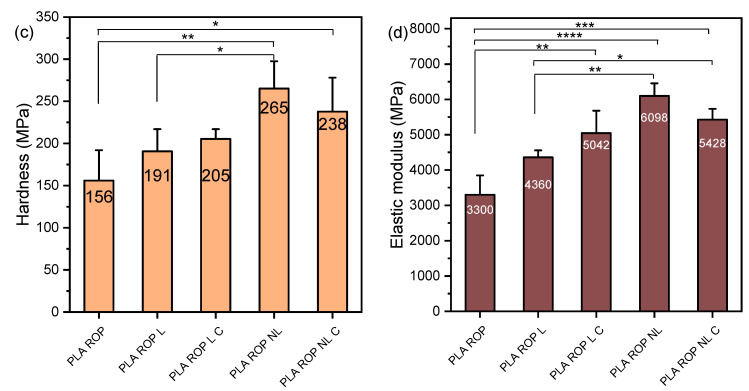
Hardness and elastic modulus of (**a**,**b**) PLA 2003D and (**c**,**d**) PLA ROP with kraft lignin and nanolignin. One-way ANOVA, post hoc Tukey HSD test * *p* = 0.01–0.05, ** *p* = 0.001–0.01, *** *p* = 0.0001–0.001, **** *p* < 0.0001.

The nanomechanical properties of the composites were examined with nanoindentation, and the force-depth curves are shown in Figure S5, while the hardness and elastic modulus values are presented in Figure 5. The nanoindentation elastic modulus of PLA 2003D and its composites with L and NL (Figure 5b) was very similar to the tensile one, and the trend was the same, i.e., both additives reduced it and made PLA softer, as witnessed by the reduction in hardness (Figure 5a). Interestingly, NL decreased both values more than L. As NL has a large free surface and subsequently more -OH groups, the interactions between the particles could be stronger and cause more aggregation during melt mixing, so the beneficial effects of the size reduction could not be harnessed. When looking at the nanoindentation elastic modulus and hardness of PLA ROP composites with N and NL in Figure 5c,d, a completely different trend is observed. Both values increased after the addition of either N or NL, with NL giving the stiffer composite. The effect of size reduction was obvious since there is a statistically significant difference in hardness and modulus between the composites PLA ROP L and PLA ROP NL. The addition of a larger amount of catalyst did not have an important effect on either value, but a decreasing trend was observed from PLA ROP NL to PLA ROP NL C. Overall, the composite PLA ROP NL stands out as it reached an E of ~6000 MPa with only 0.5 wt% of NL. The significant improvement in stiffness of the composites prepared with in situ ROP instead of melt mixing is due to the extensive interactions between the polymer and the fillers, facilitated by the initiation of ROP by the -OH groups of the lignins, resulting in the formation of PLA molecules attached to lignin. This mechanism helps disperse the polymer and prevents aggregation, ultimately giving polymer composites improved mechanical properties [21].

### 3.5. Antioxidant Activity

**Figure 6:**
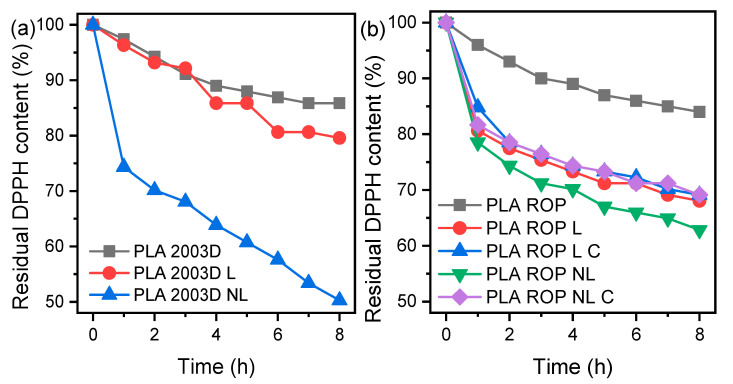
Reaction kinetics of the free radical DPPH during immersion of PLA lignin and nanolignin composites prepared by (**a**) melt compounding and (**b**) ROP.

Since lignin contains phenolic hydroxyls, it acts as a radical scavenger, which is especially useful in food packaging and biomedical applications. The radical scavenging activity of the composites was evaluated by measuring the absorption decay at 517 nm every hour for 8 h via UV-Vis. Figure 6 shows the residual DPPH content over time for PLA L and NL composites prepared by (a) melt compounding and (b) ROP during immersion in a DPPH/ethanol solution. Both PLA 2003D and PLA ROP showed negligible antioxidant activity. In the case of composites prepared by melt compounding, PLA NL composite showed higher antioxidant capacity compared to PLA L, with 50% residual DPPH content after 8 h. Similar results were reported in our previous study, where it was shown that size reduction of the lignin particles yielded stronger antioxidant activity for quite small lignin contents (up to 5 wt%) [28]. In that study, the NL used was significantly less effective in imparting antioxidant activity to PLA at 0.5 wt%, likely because the masterbatch preparation with solution casting led to the formation of some hydrogen bonds between the phenolic -OH of lignin and the polar groups of PLA. Regarding the composites prepared by ROP, all samples showed similar antioxidant activity. More specifically, PLA ROP L, PLA ROP L C, PLA ROP NL, and PLA ROP NL C yielded 68%, 69%, 63%, and 69% residual DPPH content reduction after 8 h, respectively. Among them, NL was the most effective. When 1 and 3 wt% NL with particle size ~50 nm were added to PLA with melt mixing after preparing a masterbatch, the residual DPPH content after 24 h was 92% and 80%, respectively, which is again lower than the values reported herein for either PLA 2003D or PLA ROP composites [45]. Overall, the ROP composites were less effective than the melt mixed ones, but they were still quite effective in scavenging radicals. Consuming the -OH of lignins with ROP or other modifications had a negative effect on the radical scavenging ability [21,42], so care must be taken when modifying lignin in order to achieve the optimum dispersion and at the same time maintain its antioxidant properties.

### 3.6. Optical Properties

**Figure 7:**
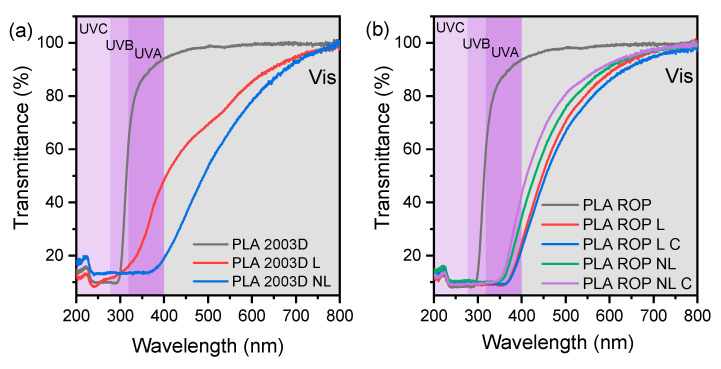
Optical properties of PLA lignin and nanolignin composites prepared by (**a**) melt compounding and (**b**) ROP.

Most commercial plastics contain UV-blocking agents for packaging applications. The surface functional groups of lignins such as phenols, ketones, and chromophores enhance the UV absorbance of PLA composites. Figure 7 shows the UV-Vis transmittance of the PLA L and NL composites prepared by (a) melt compounding and (b) ROP between 200 and 800 nm. It is important to distinguish the different UV regions, as polymer photodegradation in natural lighting is facilitated by UVB, but artificial UVC that is often used for sterilization can cause a moderate decrease in the mechanical properties of PLA [46].

PLA 2003D and PLA ROP films were transparent in the visible region and started to absorb in the UV region, below ~300 nm. PLA 2003D L and PLA 2003D NL had decreased transparency when compared to the neat polymer because of the brownish color of lignin, so their transmittance is smaller than that of PLA 2003D in the visible and UVA ranges. However, NL has a bigger slope than L, leading to lower transmittance at higher wavelengths. This is attributed to the smaller particle size of NL combined with poor dispersion in the polymer matrix [42,47].

Similarly, all the composites prepared by in situ ROP (Figure 7b), absorbed in the visible region of the spectrum. Additionally, compared to PLA 2003D and PLA ROP, the UV transmittance was lower in all composites, especially in the UVB and UVC regions. This is due to the presence of lignin and its chromophore nature. Both L and NL composites showed similar performance in blocking UV light when added with ROP in comparison with melt compounding.

### 3.7. Water Contact Angle

**Figure 8:**
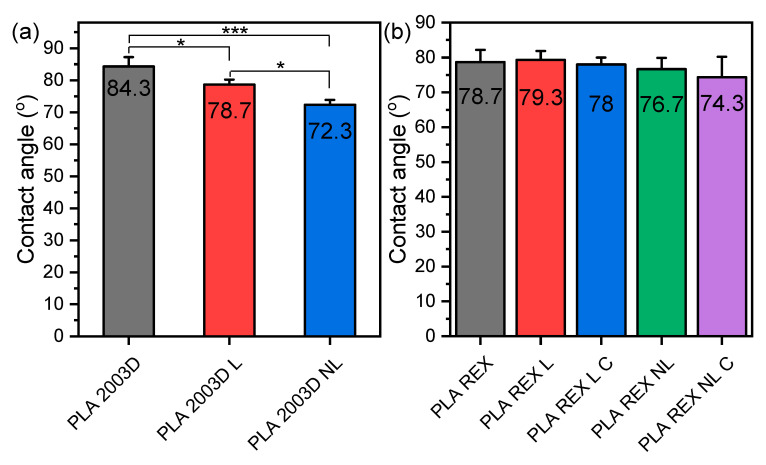
Water contact angle of PLA lignin and nanolignin composites prepared by (**a**) melt compounding and (**b**) ROP. One-way ANOVA, post hoc Tukey HSD test * *p* = 0.01–0.05, *** *p* = 0.0001–0.001.

The changes in the hydrophilicity of PLA after the addition of L and NL were evaluated by measuring the water contact angle, and the average values are shown in Figure 8. PLA ROP is slightly more hydrophilic than PLA 2003D due to its larger M_n_. The surfaces of the composites prepared by melt mixing became more hydrophilic since their water contact angle was reduced (Figure 8a) [28], and the reduction was bigger in the case of NL because of its larger specific surface and thus more accessible free -OH groups. The lignins were used as received and contained free -OH groups that increased hydrophilicity. On the other hand, the composites prepared by in situ ROP did not show any significant reduction of their contact angle, which can be attributed to the covalent bonds formed between the lignin particles and PLA during their synthesis.

## 4. Conclusions

Poly(lactic acid) composites with 0.5 wt% lignin and nanolignin were prepared with two different approaches: the typical melt mixing method and the new, faster method of in situ reactive processing. Both lignin and nanolignin reduced the UV transmittance and improved the antioxidant activity of PLA. Comparing the characteristics of the resulting composites showed that the dispersion, crystallization, mechanical, and optical properties were improved in the case of ROP-prepared samples. These improvements were attributed to the better dispersion of L and NL via ROP, which was facilitated by the formation of interfacial covalent bonds. Lignins are polyols with high functionality and act as macroinitiators in the ROP of lactide, yielding the grafting of PLA chains on lignin particles. The larger catalyst amount successfully decreased polymerization time while retaining the achieved intrinsic viscosity and molecular weight. The size reduction of lignin towards nanolignin had a significant beneficial effect on the cold crystallization and nanomechanical properties of PLA, with a maximum elastic modulus of ~6000 MPa, showcasing the importance of lignin’s size on the properties of composites synthesized with in situ ROP.

## Figures and Tables

**Table 1:** List of samples prepared and their abbreviations.

Abbreviation	Explanation
PLA 2003D	Neat PLA 2003D
PLA 2003D L	PLA 2003D with 0.5 wt% lignin prepared by melt compounding
PLA 2003D NL	PLA 2003D with 0.5 wt% nanolignin prepared by melt compounding
PLA ROP	Neat PLA prepared by in situ ROP with reactive processing
PLA ROP L	PLA with 0.5 wt% lignin prepared by in situ ROP with reactive processing
PLA ROP L C	PLA with 0.5 wt% lignin prepared by in situ ROP with reactive processing using × catalyst
PLA ROP NL	PLA with 0.5 wt% nanolignin prepared by in situ ROP with reactive processing
PLA ROP NL C	PLA with 0.5 wt% nanolignin prepared by in situ ROP with reactive processing using × 2 catalyst

**Table 2:** Free lactide content (NMR), intrinsic viscosity, average molecular weight, and polydispersity (GPC) of the studied polymers.

Sample	Free Lactide (%)	[η] (dL/g)	M_n_ (g/mol)	PDI
PLA 2003D	-	1.79	72,000	2.42
PLA 2003D L	-	1.63	82,800	2.47
PLA 2003D NL	-	1.64	62,500	2.58
PLA ROP	5.7	1.75	64,000	2.73
PLA ROP L	2.9	1.58	77,600	3.56
PLA ROP L C	3.9	1.63	66,700	3.84
PLA ROP NL	3.9	1.57	292,800	1.28
			31,700	2.26
PLA ROP NL C	3.9	1.59	273,400	1.31
			32,300	2.09

**Table 3:** DSC characteristics of the PLA lignin and nanolignin composites obtained from heating at a rate of 20 °C/min after quenching.

Sample	T_g_ (°C)	T_cc_ (°C)	T_m_ (°C)	ΔH_cc_ (J/g)	ΔH_m_ (J/g)
PLA 2003D	59.5	127	149.4	4.1	4.2
PLA 2003D L	59.9	126.6	149.3	3.8	3.9
PLA 2003D NL	59.5	126.3	149.7	3.7	3.8
PLA ROP	43.0	97.0	154.1	35.6	36.4
PLA ROP L	51.9	98.4	163.8	30.1	38.3
PLA ROP L C	51.5	99.1	163.1	31.2	36.7
PLA ROP NL	50	102.5	161.8	36	39.4
PLA ROP NL C	49	95.4	159.1	39.7	40.8

## Data Availability

All the data of this study is included in the manuscript and Supplementary Material.

## Supplementary Materials

The following supporting information can be downloaded at: https://www.mdpi.com/article/10.3390/polym15102386/s1, Figure S1: Particle size distribution curves of kraft lignin and nanolignin; Figure S2: GPC chromatographs of PLA and its composites with lignin and nanolignin prepared by reactive processing; Figure S3: ^1^H NMR spectra of ROP PLA with 0.5% (a) lignin and (b) nanolignin; Figure S4: Cooling DSC scans of the PLA ROP composites (rate 10 °C/min); Figure S5: Force-depth curves of PLA composites with lignin and nanolignin prepared by (a) melt compounding and (b) reacting processing.
