# Supplementary material for: Poly(Lactic Acid) Composites with Lignin and Nanolignin Synthesized by In Situ Reactive Processing
Source: Polymers (Basel). 2023 May 19;15(10):2386. doi: 10.3390/polym15102386 (PMC10221996; doi:10.3390/polym15102386)
Supplement: Supplementary file 1 [file polymers-15-02386-s001.zip › polymers-2405546-supplementary.pdf]

polymers

MDPI

Supplementary Materials

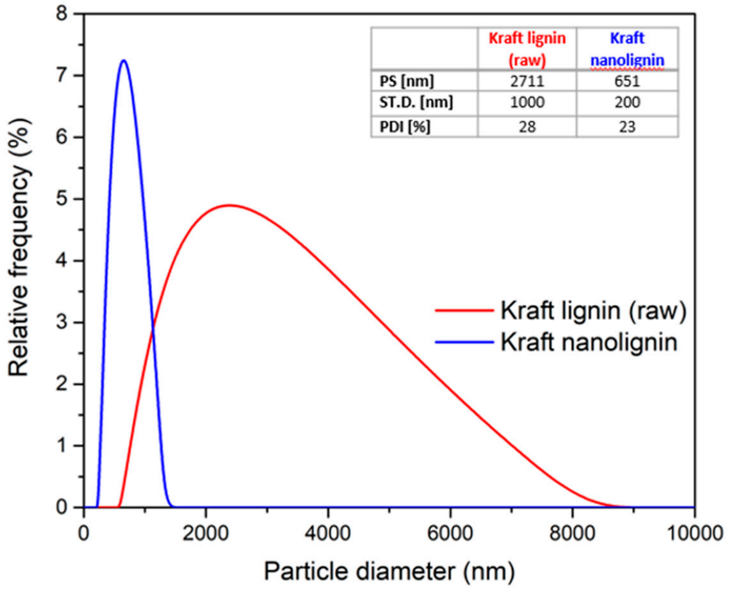

(a)

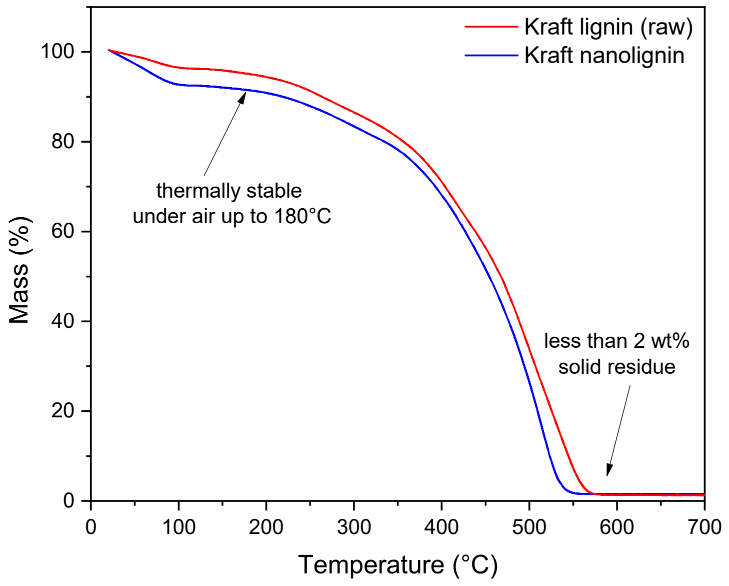

(b)

Figure S1. (a) Particle size distribution curves and (b) TGA curves of kraft lignin and nanolignin.

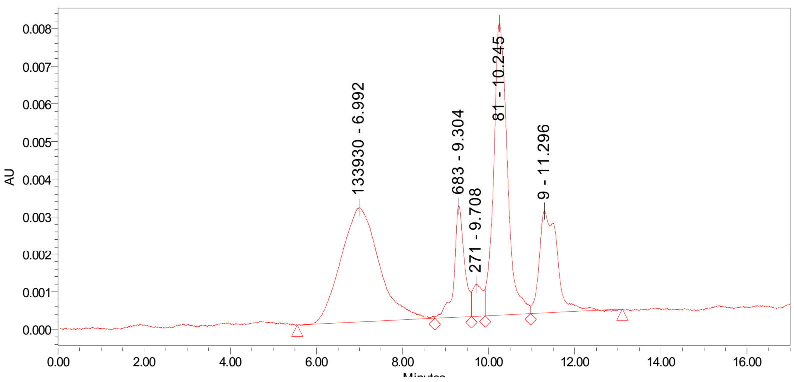

PLA ROP

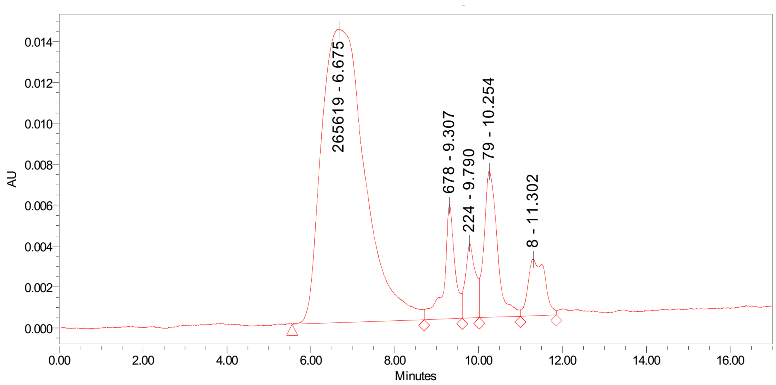

PLA ROP L

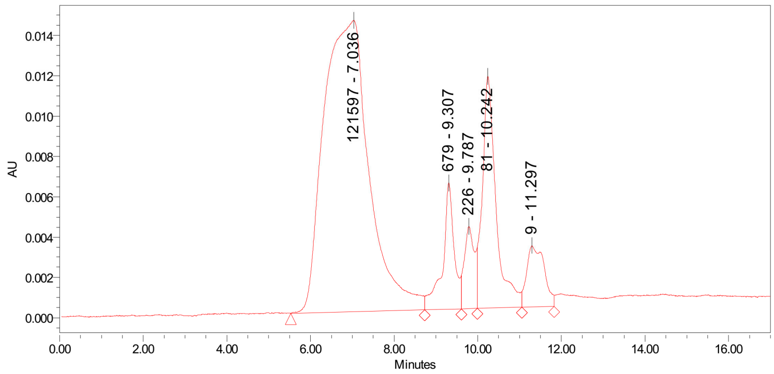

PLA ROP L C

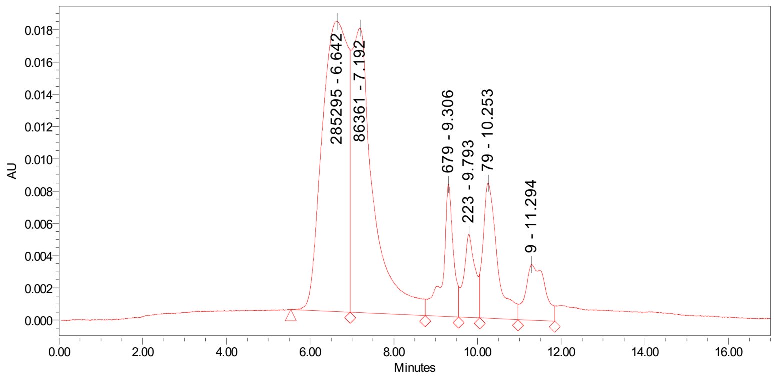

PLA ROP NL

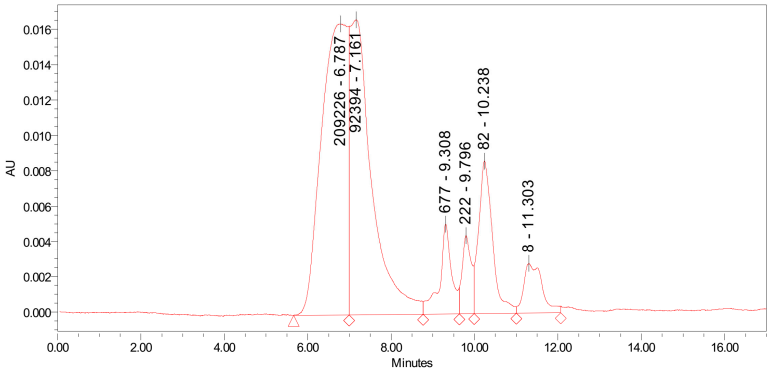

PLA ROP NL C

Figure S2. GPC chromatographs of PLA and its composites with lignin and nanolignin prepared by reactive processing.

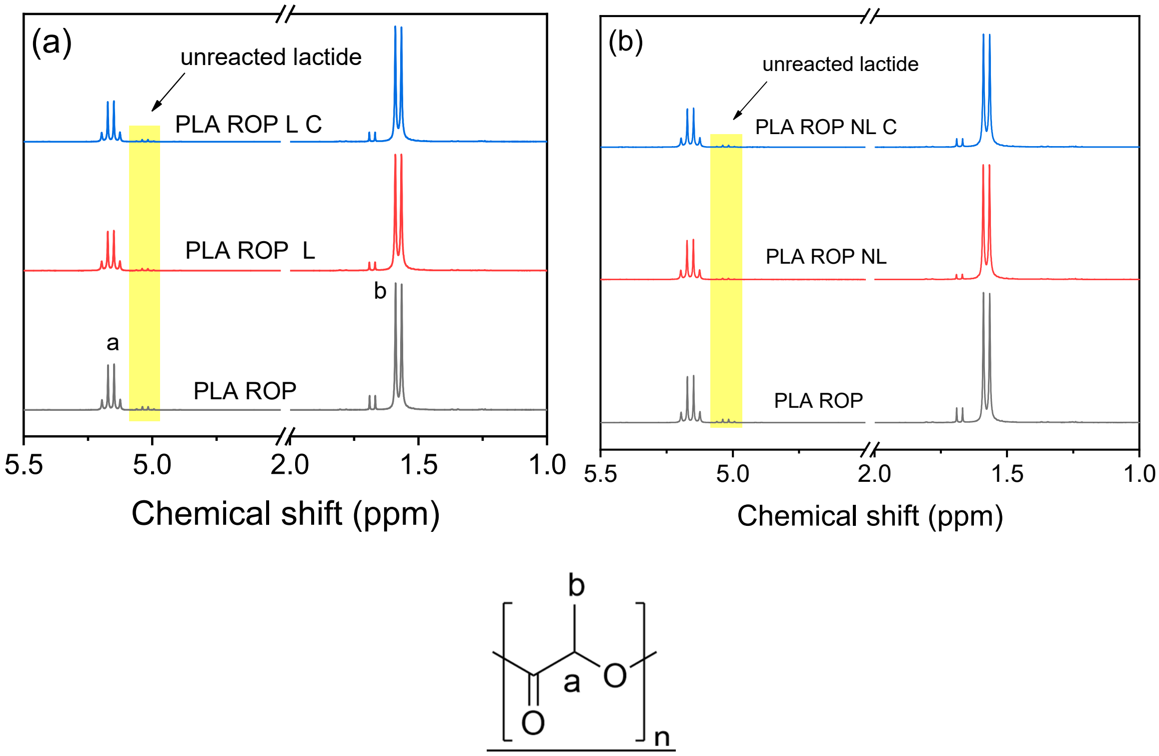

Figure S3. ^{1}H NMR spectra of ROP PLA with 0.5% (a) lignin (b) nanolignin, (c) peak assignements.

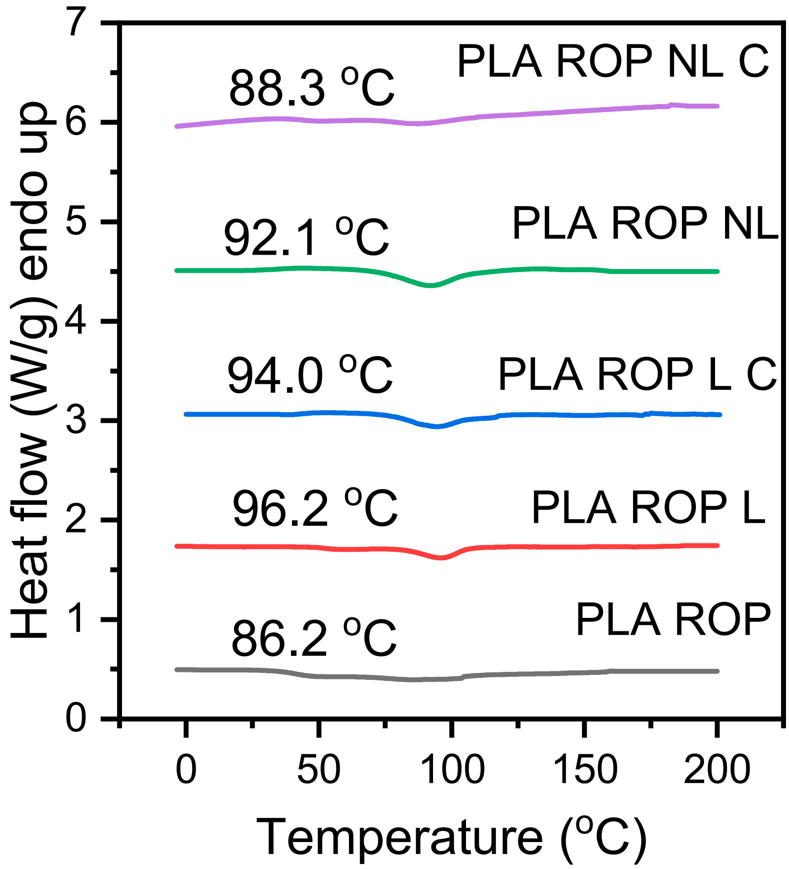

Figure S4. Cooling DSC scans of the PLA ROP composites (rate 10 °C/min).

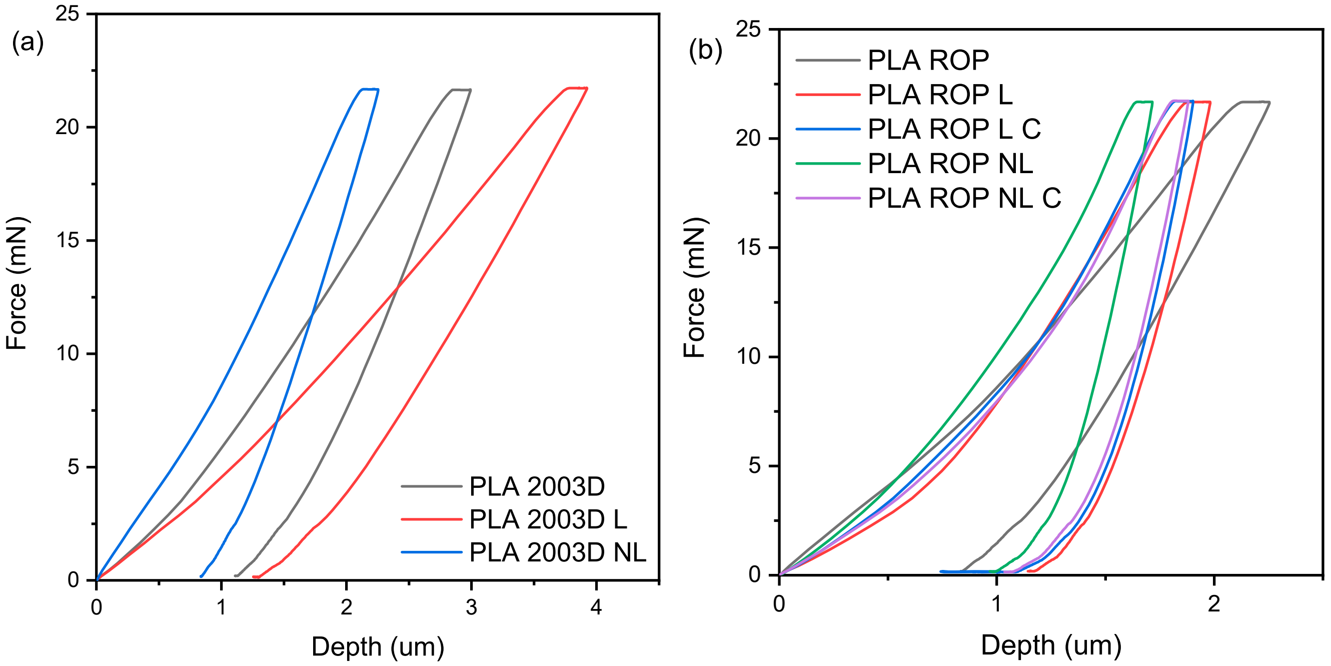

Figure S5. Force-depth curves of PLA composites with lignin and nanolignin prepared by (a) melt compounding and (b) reacting processing.
